# Effect of a Craniosacral Therapy Protocol in People with Migraine: A Randomized Controlled Trial

**DOI:** 10.3390/jcm11030759

**Published:** 2022-01-30

**Authors:** Elena Muñoz-Gómez, Marta Inglés, Marta Aguilar-Rodríguez, Sara Mollà-Casanova, Núria Sempere-Rubio, Pilar Serra-Añó, Gemma V. Espí-López

**Affiliations:** 1 Department of Physiotherapy, Faculty of Physiotherapy, University of Valencia, 46010 Valencia, Spain; elena.munoz-gomez@uv.es (E.M.-G.); marta.ingles@uv.es (M.I.); marta.aguilar@uv.es (M.A.-R.); sara.molla@uv.es (S.M.-C.); nuria.sempere@uv.es (N.S.-R.); gemma.espi@uv.es (G.V.E.-L.); 2 Research Unit in Clinical Biomechanics (UBIC), Department of Physiotherapy, Faculty of Physiotherapy, University of Valencia, 46010 Valencia, Spain

**Keywords:** migraine, physiotherapy, manual therapy

## Abstract

*Background*: Migraine is a common neurological disorder, and it is the second leading cause of disability worldwide. Manual techniques based on physical therapy have been proposed to improve migraine aspects; however, further research is needed on their effectiveness. The aim of this study was to evaluate the effectiveness of a craniosacral therapy protocol on different features in migraine patients. *Methods*: Fifty individuals with migraine were randomly divided into two groups (*n* = 25 per group): (i) craniosacral therapy group (CTG), following a craniosacral therapy protocol, and (ii) sham control group (SCG), with a sham treatment. The analyzed variables were pain, migraine severity and frequency of episodes, functional, emotional, and overall disability, medication intake, and self-reported perceived changes, at baseline, after a 4 week intervention, and at 8 week follow-up. *Results*: After the intervention, the CTG significantly reduced pain (*p* = 0.01), frequency of episodes (*p* = 0.001), functional (*p* = 0.001) and overall disability (*p* = 0.02), and medication intake (*p* = 0.01), as well as led to a significantly higher self-reported perception of change (*p* = 0.01), when compared to SCG. In addition, the results were maintained at follow-up evaluation in all variables. *Conclusions*: A protocol based on craniosacral therapy is effective in improving pain, frequency of episodes, functional and overall disability, and medication intake in migraineurs. This protocol may be considered as a therapeutic approach in migraine patients.

## 1. Introduction

A migraine is a primary headache, and it is one of the major leading causes of disability in people under the age of 50 [1,2]. Migraine constitutes a complex brain network disorder with a strong genetic basis that involves multiple subcortical, cortical, and brainstem regions [3]. Moreover, patients with migraine may present musculoskeletal dysfunctions [4], which in turn facilitate the development of migraine [5]. Furthermore, there are other types of alterations that can mediate the generation of migraines, such as certain emotional disorders [6,7]. Indeed, emotional stress and negative emotional events have been shown to play an important role in precipitating or exacerbating migraine attacks [7].

The most common preventive and symptomatic treatment for migraine is pharmacological. However, this type of treatment involves some side-effects, such as gastrointestinal, cardiovascular, and central nervous system complications [8]; hence, other treatments may be an alternative, such as psychological treatment, patient education, acupuncture, supervised physical activity, and manual techniques (i.e., chiropractic treatment and physiotherapy) [9,10,11,12].

Regarding physiotherapy, some studies have suggested the effectiveness of manual techniques in individuals with migraine, specifically on pain intensity, number of days, duration of the episodes, disability, and medication intake [11,13,14]. Craniosacral therapy has also been used to treat headaches and migraines [15,16]. This therapy is characterized by being a set of noninvasive fascial techniques performed between the skull and the sacrum [17], whose objective is to relax myofascial structures and normalize sympathetic nerve activation, often increased in patients with chronic pain [18,19], thus improving body function [20]. Some studies have suggested positive effects of craniosacral therapy on pain intensity and frequency [15,21,22], disability [15,21,22,23], quality of life [15,21], medication intake [22], treatment credibility, and satisfaction [24] in migraine patients. However, the quality of previous studies hampers the possibility to determine the magnitude of this effect [25]. In addition, some studies have reported controversial results on the impact of migraine [25].

Furthermore, to date, no study has analyzed the effectiveness of this type of intervention on emotional disability in migraine patients. In this regard, it has been reported that migraine patients, unlike patients with other types of headaches or pain disorders, exhibit hypersensitivity to somatosensory stimuli, which may be due to an altered perception and cerebral processing of somatosensory stimuli [26]. Altered sensory processing has in turn been related to neurobiological differences [27] and can lead to disruptions in other cognitive domains, such as emotional processing [28]. In this context, the study of the effectiveness of these nonpharmacologic interventions on emotional-related variables in migraine sufferers becomes particularly important, in order to avoid their negative consequences.

Thus, the objective of this study was to evaluate the effect of a craniosacral therapy protocol on pain intensity, migraine severity and frequency, emotional, functional, and overall disability, self-reported perceived change, and medication intake after treatment in people with migraine, compared to a placebo treatment. Furthermore, the medium-term effects of the treatments on the assessed variables were assessed.

## 2. Materials and Methods

### 2.1. Recruitment and Participants

Fifty people diagnosed with migraine participated in the study. They were recruited from primary care centers in Valencia (Spain) in July 2018. Inclusion criteria were as follows: (i) individuals aged 18–50 years; (ii) diagnosed according the International Headache Society (IHS) criteria [29]; (iii) four or more episodes per month; (iv) more than 1 year history of migraine; (v) current acute and prophylactic migraine medication regimens being stabilized for 4 weeks prior to enrolment. Patients were excluded in case of concomitant tension-type headaches or other headaches, signs of vertebral artery or internal carotid artery commitment, temporomandibular disorders, spinal radiculopathy, decompensated blood pressure, vertigo, depression, or pregnancy (or pregnancy intention). Lastly, patients were to not have received any previous manual therapy treatment for migraine.

### 2.2. Study Design

This was a CONSORT-compliant randomized single-blind controlled trial (within a broader project, registration number NCT03555214). The sample was randomly divided into two groups: (a) craniosacral therapy group (CTG) (*n* = 25), and (b) sham control group (SCG) (*n* = 25). Both treatments lasted 4 weeks and included four sessions (one per week). Patients were assessed pre-intervention (T1), post-intervention (T2), and at a 1 month post-intervention follow-up (T3).

Signed written informed consent was obtained from all participants prior to participation in the study. All procedures were conducted in agreement with the World Medical Association Declaration of Helsinki principles. Lastly, all protocols were approved by the Ethics Committee of the University of Valencia (H1509655117217).

### 2.3. Randomization, Blinding, and Masking

Patients and statisticians were blinded to treatment allocations. Blinding was maintained and ensured until the completion of the entire study by avoiding any information regarding study hypothesis, details of interventions, random assignment, outcome measures, and outcome analysis. The randomization method consisted of a computer-generated random sequence table with a non-balanced three-block design (GraphPad Software, Inc., San Diego, CA, USA).

### 2.4. Interventions

All interventions were applied at the same time of day and in the same room, trying to standardize the treatment, and we tried to mimic the environment of a typical physiotherapy intervention (i.e., clinical tests, durations, and resources). The interventions were carried out with the patient in supine decubitus. The CTG received a manual therapy treatment focused on the craniosacral region including five techniques (Appendix A), and the SCG received a hands-on placebo intervention. After the intervention, individuals remained in supine with a neutral neck and head position for 10 min, to relax and diminish tension after treatment [30]. The techniques were executed by the same experienced physiotherapist in both groups. Participants were asked to report any side-effects during or after the intervention.

#### 2.4.1. Craniosacral Therapy Group

Techniques were applied in each session according to the following predefined sequence:

Suboccipital inhibition technique. Both hands were placed under the occiput, with the fingers in contact with the atlas (posterior arch). Deep, sliding, and progressive pressure was applied for 10 min [31]. The objective of this technique was to relax the suboccipital muscles [32].

Frontal technique. The therapists’ ring and little fingers were placed along the outside of the frontal bone (zygomatic processes), while the middle and index fingers were positioned next to the frontal bone (midline). A slight pressure in a posterior direction was performed with the index fingers on the midline of the frontal bone, and, at the same time, the ring fingers were moved in an anterior and caudal direction for 5 min [33]. The aim of this technique was to relax the tissue around cranial structures [33], since extracranial tissues such as pericranial muscles and periosteum are innervated by some meningeal afferents, and such tissues may be related to migraine onset [34].

Sphenoid technique. The index finger was put over the sphenoid (greater wing), the middle finger on the pterion, the ring finger behind the ear over the asterion, and the little finger over the occiput (lateral angle). Both thumbs were applied together on the midline of the head. A gentle distraction force was performed for 5 min [35]. The objective of this technique was to relax the tissue around the cranial structures [35].

Fourth ventricle technique. Both hands with palms up were applied under the patient’s occiput, with the thumb tips together. The therapist made a slight approximation of the thenar eminence and a cephalic traction for 10 min [36]. This cranial technique may be helpful in cases of imbalance in the autonomic nervous system [37] and may accordingly provide analgesia and reduce pain sensitivity [38].

Lumbosacral technique. One flat and palm-up hand was located under the sacrum and the lumbar vertebrae L4–L5, whereas the other hand was placed flat and palm down on the pelvic upper surface, with both hands vertically aligned. The therapist performed a slight compression with both hands for 5 min [23]. The objective of this technique was to relax the muscles and other structures around the lumbosacral area to improve their movement and to improve the sagittal balance of the spine, since there are significant correlations between occipitocervical and spinopelvic alignment [39].

#### 2.4.2. Sham Control Group

Placebo intervention. A hands-on placebo superficial contact was performed by placing both hand palms under the occiput for 10 min, without touching the suboccipital muscles. No force, pressure, or movement was performed [36,38].

### 2.5. Assessments

Pain, migraine severity, frequency of the episodes, and functional, emotional, and overall disability were assessed at baseline (T1), at 4 weeks (T2), and at 8 weeks (T3) after the intervention. Medication intake reduction and self-reported perceived changes after treatment were recorded only at T2 and T3.

Pain. Pain intensity was assessed using the Visual Analog Scale (VAS) [40], whereby patients rated their perceived pain intensity level on a horizontal 10 cm line, where 0 = “absence of pain” and 10 = “worst pain imaginable”. It is considered a valid and reliable instrument, with an ICC = 0.97 (95% CI = 0.86–0.98) [40].

Migraine severity and frequency of the episodes. The migraine severity (i.e., mild, moderate, and severe) and frequency of the episodes (i.e., once a month, 2–4 times a month, and once a week) were assessed responding to the relevant questions of the Headache Disability Index (HDI) [41]. HDI is a valid and reliable tool [41] that is widely accepted for assessing the effectiveness of nonpharmacological interventions in frequent episodic or chronic migraine [42], which covers the endpoints recommended by the IHS.

Functional emotional and overall disability. They were assessed using the HDI [41], which evaluates the migraine-induced disability in daily life. It includes 25 items that can be divided into a 12-item functional subscale (i.e., functional disability) and a 13-item emotional subscale (i.e., emotional disability). Each item has three possible answers (no = 0 points, sometimes = 2 points, yes = 4 points). The total score (i.e., overall disability) ranges from 0 = “no disability” to 100 = “maximum disability”. The Cronbach alpha reliability is α = 0.76 for the functional subscale, α = 0.82 for the emotional subscale, and 0.83 for the total score [41].

Medication intake. Symptomatic medication intake was registered in a standardized migraine diary, which also included information about migraine days, intensity (VAS scale), and severity (severe, moderate, or mild), so that the patients would remember these data for post-treatment evaluation. This variable was registered as the number of pills per day. The percentage of medication intake reduction was calculated as previously described [43].

Self-reported perceived change after treatment. This was evaluated by means of the Patients’ Global Impression of Change (PGIC) scale, consisting of a verbal scale, with seven points: “very much improved”, “much improved”, “minimally improved”, “no change”, “minimally worse”, “much worse”, and “very much worse” [44]. This scale has been previously used in chronic pain individuals [44] and has shown an excellent retest reliability (ICC = 0.90) [45].

### 2.6. Sample Size Calculation

Sample size was computed taking into consideration that our study included two groups (i.e., control and experimental groups), and that three measurements were conducted. We set a power of 80% and an effect size of *d* = 0.88 according to a previous study conducted by Espí-López et al. [31]. With this consideration, a minimum sample size of 24 participants (i.e., 12 participants per group) was required. However, the recruitment was doubled (i.e., 50 participants) taking into consideration possible dropouts.

### 2.7. Data Collection and Statistical Analyses

All statistical analyses were performed with SPSS v24 (IBM SPSS, Inc., Chicago, IL, USA). Standard statistical methods were used to obtain the mean and standard deviation (SD).

For the analysis of continuous variables measured three times (i.e., pain, emotional disability, functional disability, and overall disability), a two-factor mixed multivariate analysis of variance (MANOVA) was conducted with a between-subject factor “treatment group” having two categories (i.e., SCG and CTG) and a within-subject factor “time measurements” having three categories (i.e., T1, T2, and T3). Post hoc multiple comparisons were conducted using a single-step procedure, i.e., the Bonferroni method, which adjusts type I error based on the *t*-distribution (α/number of comparisons). We evaluated the assumption of homoscedasticity using Levene’s test and of sphericity using Mauchly’s test. For the categorical variables measured three times (i.e., Migraine severity and frequency of the episodes), the relationship between the categories of each variable and the time measurement (T1, T2, and T3), for each group, was explored using the chi-square test. Furthermore, the relationship between the categories of the variables and the groups were also explored using the chi-square test for each time measurement.

To analyze the effect of the treatment on the medication intake, a two-factor mixed MANOVA with the between-subject variable “group” and the within-subject variable “time” (i.e., T2 and T3) was used. A chi-square test was used for the analysis of the self-reported perceived change after treatment to evaluate the statistical differences between groups and between time measurements. Furthermore, to explore the similarity between groups at baseline, the chi-square test was used for the categorical variables and one-way ANOVA was used for the continuous variables. The α level was set below 0.05 for all tests. The effect size of all continuous variables was computed by Cohen’s d, thus rating the effect size as follows: large (>0.80), medium (0.50–0.80), or small (0.20–0.50), [46]. For categorical variables, the effect size was reported by the contingency coefficient (CC).

## 3. Results

### 3.1. Participants

**Figure 1:**
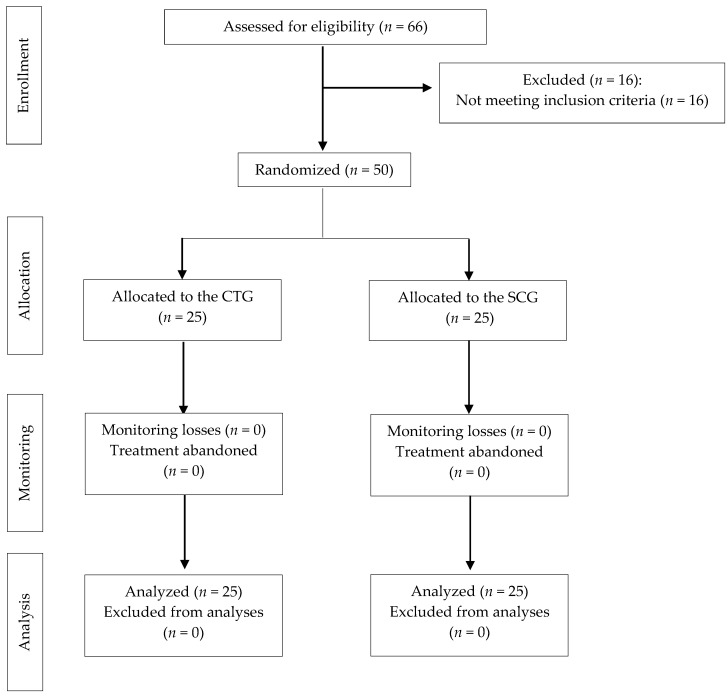
Flowchart according to CONSORT Statement for the Reporting of randomized trials. CTG: craniosacral therapy group; SCG: sham control group.

Sixty-six individuals were assessed for eligibility, but 16 failed to meet the inclusion criteria; thus, 50 people (40 women and 10 men) were randomized, and all of them completed the study (25 in CTG and 25 in SCG). Following the CONSORT guidelines, Figure 1 presents a flow diagram for this trial [47]. So that there were no dropouts, the sample was selected well, requesting adherence commitment, unless an adverse event arose due to the treatment or for other reasons; in addition, a rigorous follow-up of the subjects was carried out, which is why all subjects were able to comply with the treatment without dropouts.

The mean (SD) age of the participants was 40.1 (9.9) years. Table 1 shows the baseline demographic and migraine characteristics. There were no significant baseline differences between groups in any variable (*p* ≥ 0.05). Regarding intervention-related side-effects, some participants (*n* = 5) reported a slight dizziness lasting seconds to a few minutes when getting up from the stretcher, but no serious side-effect was reported.

### 3.2. Effect of the Treatment on Pain and Disability

A significant interaction between factors “groups” and “intervention measurements” in total HDI F (2, 96) = 3.23, *p* < 0.05, η^2^ = 0.06, and in functional HDI F (2, 96) = 6.15, *p* < 0.05, η^2^ = 0.11, but not in pain and emotional HDI (*p* ≥ 0.05), was found.

The results of pairwise comparisons are shown in Table 2. When analyzing the effect of the treatment in each group and between groups, pain intensity was significantly reduced in T2 and T3 in the CTG compared to SCG. With regard to migraine-induced disability, there was a significant reduction in the values both on the functional subscale and in the global assessment after treatment, and such reduced scores were maintained at T3, as can be seen when comparing groups in T2 and T3.

### 3.3. Effect of the Treatment on Migraine Severity and Frequency of the Episodes

**Figure 2:**
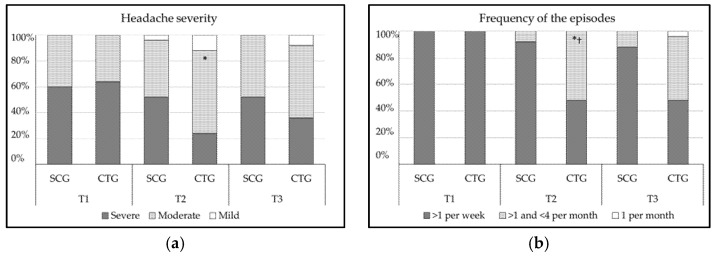
(**a**) Percentage of participants who rated each category of the migraine severity of the Headache Disability Index (HDI; (**b**) percentage of participants who rated each category of the frequency of episodes of the Headache Disability Index (HDI). CTG: craniosacral therapy group; SCG: sham control group; T1: pre-treatment; T2: post-treatment; T3: follow-up; * *p* < 0.05 vs. T1; ^†^ *p* < 0.05 vs. SCG.

As a result of the chi-square tests, the SCG showed no significant relationship between migraine severity and the measurements conducted (*p* ≥ 0.05; data not shown). Nevertheless, the CTG presented a significant moderate improvement at T2 (χ^2^ (2) = 9.51, *p* = 0.009, CC = 0.40) as can be observed in Figure 2a. There were no significant differences between scale categories and groups at T2 or at T3 (*p* ≥ 0.05, respectively).

Similarly, there was no relationship between categories and measurement times in migraine frequency in the SCG (*p* ≥ 0.05; data not shown). However, when the CTG was analyzed, the results presented a significant moderate improvement at T2 (χ^2^ (1) = 17.57, *p* < 0.001, CC = 0.51) and at T3 (*χ*^2^ (2) = 17.57, *p* < 0.001, CC = 0.51), as noted in Figure 2b. In addition, in migraine frequency, there were significant differences between scale categories and groups at T2 (χ^2^ (1) =11.52, *p* < 0.001, CC = 0.43), and at T3 (χ^2^ (2) = 9.34, *p* < 0.01, CC = 0.40).

### 3.4. Effect of the Treatment on Medication Intake

Of the 50 individuals participating in the study, one participant did not take preventive or symptomatic medication at any time during the study; accordingly, this assessment was performed on 49 individuals. Table 2 shows that the drop in medication intake was significantly greater in CTG than in SCG at T2 *t*(47) = 2.62, *p* < 0.05, *r* = 0.35) and at T3 *t*(47) = 2.47, *p* < 0.05, *r* = 0.33).

### 3.5. Self-Reported Perceived Change after Treatment

Table 3 shows the results of the PGIC scale for the SCG and the CTG at T2 and T3 and the statistical results from the analysis of the association between scale categories and time measurements in each group.

A significant moderate association between scale categories and groups was found, both at T2 (χ^2^ (5) =13.23, *p* < 0.01, CC = 0.46) and at T3 (χ^2^ (4) = 20.14, *p* < 0.001, CC = 0.54), showing a higher proportion of participants in positive categories (i.e., much improved and very much improved) in the CTG compared to the SCG. Furthermore, only CTG showed a significant association between time measurements and categories, with the number of people in positive categories decreasing at T3 compared to T2.

## 4. Discussion

The results of the present study show that a craniosacral therapy protocol reduces pain, migraine severity, frequency of attacks, functional disability, emotional disability, overall disability, and medication intake in migraine patients. To the best of our knowledge, this is the first study to evaluate the therapeutic effects achieved following this manual therapy protocol on emotional disability in patients with migraine by applying different techniques on the cranial sphere and sacral region, techniques previously used independently in other studies.

Craniocervical and lumbosacral soft-tissue manual techniques, applied separately, both in patients with migraine and in those with tension or cervicogenic headaches, have previously shown good results in terms of pain, migraine severity, frequency of migraine episodes, overall disability, and medication intake [21,22,23,31,32,36]. However, in addition to such variables, our approach includes the emotional disability variable, an essential aspect in patients with primary headaches [48,49] and chronic pain [50].

The most studied pain-related variables in migraines are pain intensity/severity and the frequency of attacks [51]. Our results showed that CTG experienced an improvement in pain severity evaluated according to HDI. Thus, the percentage of patients experiencing severe pain at T1 dropped from 64% to 24% at T2. At T3, 56% of the patients still reported moderate pain. These results are in line with the reduction in pain intensity (VAS), which exhibited a difference with respect to the baseline measurement of 1.14 points at T2 and 1.20 points at T3 in the CTG, exceeding the minimal clinical important difference at both times [52]. Other authors reported a decrease of 1.67 points after manual therapy treatment in the orofacial and cervical region, reaching 2.25 points after 6 weeks and 3.50 points after 12 weeks [53]. Nevertheless, the results of the current study cannot be comparable with these studies, since their sample included chronic migraine patients with temporomandibular disorders, while our study excluded patients with such disorders. In terms of migraine frequency using the HDI, 52% of the CTG subjects who reported more than four migraine attacks per month at baseline experienced a reduction in frequency to less than four attacks at T2 and 48% maintained this improvement at T3; this supports the effectiveness for this variable. These results are consistent with those published by Cerritelli et al. [22], who observed that the average frequency dropped from 22.5 to 1.2 days per month after 6 months of manual therapy. However, the treatment period in their study was much longer and a protocol was not specified.

Pain, whether episodic or chronic, interferes with the patient’s life, affecting their physical and emotional condition [54,55]. On the one hand, the functional and overall disability improved at T2 by 23.21% and 23.02%, respectively, and at T3 by 21.12% and 21.12%, respectively. Likewise, previous results reported by our group showed an improvement in the overall disability collected by the MIDAS questionnaire in migraine patients treated with myofascial trigger point therapy and stretching exercises combined with the suboccipital inhibition technique [31]. However, in that case, an evaluation was only performed immediately after the intervention; thus, a longer follow-up could have determined the long-term effects of therapy. Other authors [23] reported improvements in overall disability according to the HIT-6 questionnaire, reducing the score from 62 to 58 points after an intervention using craniosacral therapy in subjects with migraine. However, a placebo group was not included in this study; hence, so improvements could not be attributed to the intervention itself, since the placebo effect of light massage was not studied. Our research shows that the improvement in both variables (i.e., functional and overall disability) was only evidenced in the manual therapy group, not in the placebo group. On the other hand, the emotional burden associated with migraine becomes especially important since it can influence prevalence, prognosis, treatment, and clinical results [56]. In fact, depression is a possible risk factor for migraine chronification [57,58] and, conversely, its approach could revert chronic migraine lasting less than 2 years back to episodic migraine [59]. In the present study, emotional disability improved significantly in the CTG only at T3, which could be due to the fact that the effect of the proposed treatment is not immediate and takes time to yield positive results for this variable [14]. However, the CTG showed a trend toward improvement at T2, with a similar magnitude of improvement (6 points) to that of T3 (5.36 points), where a significant reduction was indeed observed. In this aspect, our results cannot be compared with previous studies, since this is the first to address emotional disability in migraine patients treated with cranial, cervical, and lumbosacral soft-tissue manual therapy. D’Ippolito et al. [60] performed a retrospective review of the medical records of migraine patients treated from 2011 to 2015, and they observed a significant improvement in the level of anxiety; however, the nonrandomized selection of participants, the small sample size (*n* = 11), and the lack of a control group prevent extrapolation of the results and establishing whether the changes obtained were due to the intervention alone.

Interestingly, we observed a decrease in medication intake by 36.04% at T2 and 31% at T3 in CTG. In line with these results, other authors have observed that the overall use of analgesics, NSAIDs, and triptans was significantly lower after applying manual treatment compared to when TENS was used [61] or compared to the placebo treatment [22]. This can be explained given that the efficacy of symptomatic medication and a lower need for its use are associated with improved migraine parameters (such as the frequency of attacks) and lower emotional load [62].

The perception of change variable after a therapeutic intervention is important, since it provides clinically relevant information on the perceived effect of treatment [63]. In this regard, 52% of CTG participants at T2 and 12% at T3 felt that they had improved a fair amount or a great deal, i.e., they achieved a clinically significant improvement in this variable [64,65], consistent with the improvement of the variables previously analyzed. Other authors obtained positive results on the PGIC scale in patients with migraine [66]; however, the participants received a combined treatment of physical therapy and specific prescribed medication, while our study used only manual therapy techniques without pharmacological prescription. This implies that the changes obtained were only due to the protocol applied. Moreover, both of our study groups continued with their usual prescribed medication [51], and changes in symptomatic medication intake were likewise evaluated.

Given the possible side-effects of taking migraine drugs [8] and that migraine also tends to become chronic [57], our results showing a decrease in medication intake after the proposed treatment are especially relevant.

This study had some limitations, mainly related to the study participants’ characteristics. Firstly, we included only people suffering more than four episodes per month. Secondly, they were mostly women, which could bias the results; thus, the results are not entirely generalizable to all migraine patients. However, preventive treatments are now being considered in patients suffering from migraines ≥4 days per month, whereas people suffering one to four episodes are less prone to medication [67]. Furthermore, migraine is twice as prevalent in women as in men. In addition, the duration of treatment sessions was different between groups, but the aim of the study was to analyze the placebo effect previously described by the mere hands-on or massage factor [68]. Nevertheless, future studies including a control group without touch or other manual techniques which have been proven to be effective in other types of headaches (i.e., articulatory techniques [30]) may be of interest. Another possible limitation was the short duration of treatment or the lack of long-term follow-up; thus, we could not ascertain whether the observed beneficial the effects would remain after 2 months. Nevertheless, it is interesting to evaluate the effectiveness of short-term treatments, such as the one proposed in this article, since it facilitates adherence to treatment. Future studies addressing these issues are needed. Lastly, participants were recruited from primary care centers of one city, which may jeopardize generalizability. Thus, more studies applying this protocol in other populations and other migraine features (i.e., fewer than four episodes per month) are needed to generalize the results.

In general, all manual therapy techniques described here may be applied by different care providers, as long as they are well trained in manual therapy and craniosacral therapy. However, we recommend that these techniques are carried out by the same therapist throughout the entire treatment, in order to keep variations low (i.e., pressure, speed), to monitor the progress and to reinforce the therapeutic alliance. Furthermore, it is important to consider any possible contraindications to these techniques, such as vertebral artery or internal carotid artery commitment, spinal radiculopathy, vertigo, or decompensated blood pressure.

## 5. Conclusions

A treatment protocol based on craniosacral therapy is effective in reducing pain intensity, migraine severity, frequency of attacks, functional and emotional disability, and symptomatic medication intake, as well as improving the post-treatment perception of change in patients suffering from migraine ≥4 days per month, maintaining such changes 1 month after the intervention. This reproducible manual therapy protocol may be considered as a valid therapeutic approach in individuals with migraine.

## Figures and Tables

**Table 1:** Baseline demographic and migraine characteristics.

Variables	SCG (*n* = 25)	CTG (*n* = 25)
**Gender ^a^**		
Male	5 (20)	5 (20)
Female	20 (80)	20 (80)
**Migraine frequency ^a^**		
From 4 to 15 days per month	14 (56)	15 (60)
>15 days per month	11 (44)	10 (40)
**Medication ^a^**		
Preventive medication	1 (4)	2 (8)
Symptomatic medication	24 (96)	22 (88)
No medication	0 (0)	1 (4)
**Family history ^a^**	17 (68)	18 (72)
**Trigger factors ^a^**		
Hormonal changes	9 (36)	8 (32)
Food or drink	7 (28)	6 (24)
Stress	16 (64)	18 (72)
Fatigue, exertion	8 (32)	10 (40)
Other (change in weather, medication)	7 (28)	11 (44)
Hormonal changes	9 (36)	8 (32)
Food or drink	7 (28)	6 (24)
**Accompanying symptoms ^a^**		
Nausea/vomiting	16 (64)	12 (48)
Aura	6 (24)	4 (16)
Photophobia	15 (60)	16 (64)
Phonophobia	10 (40)	10 (40)
Age ^b^	37.64 (9.42)	40.92 (7.95)
Age of onset ^b^	19.96 (10.71)	18.72 (11.2)
Period of evolution (years) ^b^	17.68 (9.94)	22.20 (12.36)
Pain ^b^	7.68 (1.02)	7.60 (1.15)

^a^ Data shown as absolute frequency (% relative frequency); ^b^ data shown as mean (standard deviation). CTG: craniosacral therapy group; SCG: sham control group.

**Table 2:** Effect of the treatment on pain, functional, emotional, and overall disability and decrease in medication intake between measurement times and between groups.

MANOVA Interaction Results									
F(6, 190) = 2.90, *p* = 0.01, ŋ^2^ = 0.09									
		Measurement Time Comparison Mean (Standard Deviation)			Group Comparison Mean Difference (95%CI); Effect Size (*d*)		*p*-Values of the Univariate Analysis		
Variable	Group	T1	T2	T3	T2	T3	P_Time_	P_Group_	P_Time × Group_
Pain (VAS)	SCG	7.68 (1.02)	7.42 (1.57)	7.26 (1.25)	0.96 ^†^ (0.2 to 1.72); *p* = 0.01; *d =* 0.74	0.86 ^†^ (0.11 to 1.61); *p* = 0.03; *d =* 0.66	<0.01	0.02	0.05
	CTG	7.60 (1.15)	6.46 * (1.04)	6.40 * (1.38)					
Functional disability (HDI)	SCG	33.76 (7.06)	32.64 (6.63)	32.12 (5.99)	7.76 ^†^ (3.46 to 12.06); *p* = 0.001; *d =* 0.18	6.76 ^†^ (2.44 to 11.08); *p* = 0.003; *d =* 1.03	<0.01	0.01	<0.01
	CTG	32.40 (7.75)	24.88 * (8.41)	25.36 * (8.92)					
Emotional disability (HDI)	SCG	26.40 (10.65)	25.44 (9.05)	25.04 (10.71)	5.12 (−1.85 to 12.09); *p* = 0.15	4.08 (−2.5 to 10.66); *p* = 0.22	0.08	0.26	0.29
	CTG	26.32 (12.13)	20.32 (14.8)	20.96 * (12.36)					
Overall disability (HDI)	SCG	60.16 (15.87)	58.08 (14.19)	57.16 (14.71)	12.88 ^†^ (2.58 to 23.18); *p* = 0.02; *d =* 0.73	10.84 ^†^ (0.85 to 20.83); *p* = 0.03; *d =* 0.62	<0.01	0.05	0.05
	CTG	58.72 (18.32)	45.20 * (21.31)	46.32 * (20.01)					
Medication intake (%)	SCG	-	14.54 (25.17)	12.08 (27.34)	−21.5 ^†^ (−37.98 to -5.01); *p* = 0.01; *d =* −0.76	−18.92 ^†^ (−34.3 to -3.53); *p* = 0.02; *d =* −0.71			
	CTG	-	36.04 * (31.66)	31.00 * (26.18)			0.48	<0.01	0.81

Data are expressed as the mean (standard deviation). CI: confidence interval. D: Cohen’s effect size (only for the significant comparisons). CTG: craniosacral therapy group; SCG: sham control group; VAS: Visual Analog Scale; HDI: Headache Disability Index; T1: pre-treatment; T2: post-treatment; T3: follow-up. * Significant differences vs. T1 (*p* < 0.05); ^†^ significant differences between groups (*p* < 0.05).

**Table 3:** Self-reported perceived change after treatment between measurement times.

	Measurement Times			
	SCG (*n* = 25)		CTG (*n* = 25)	
	T2	T3	T2	T3
**Self-Reported Perceived Change after Treatment (PGIC Scale)**	*p* > 0.05		*χ*^2^(4) = 10.90, *p* < 0.01, CC = 0.42	
Minimally worse	2 (8)	3 (12)	0 (0)	0 (0)
No change	15 (60)	20 (80)	7 (28)	8 (32)
Minimally improved	6 (24)	2 (8)	5 (20)	14 (56)
Much improved	2 (8)	0 (0)	11 (44)	3 (12)
Very much improved	0 (0)	0 (0)	2 (8)	0 (0)

Data are shown as the absolute frequency (% relative frequency). CC: contingence coefficient (only for the significant association). CTG: craniosacral therapy group; SCG: sham control group; PGIC scale: Patient Global Impression of Change scale; T2: post-treatment; T3: follow-up.

**Figure A1:**
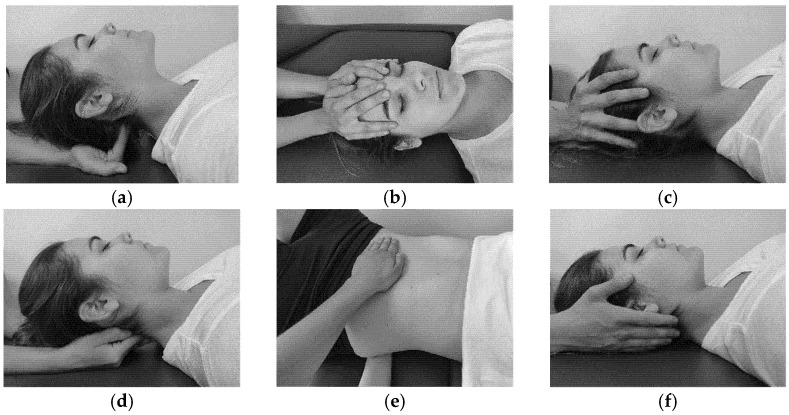
Description of manual therapy interventions: (**a**) suboccipital inhibition technique; (**b**) frontal technique; (**c**) sphenoid technique; (**d**) fourth ventricle technique; (**e**) lumbosacral technique; (**f**) placebo technique.
